# Imaging of room-temperature ferromagnetic nano-domains at the surface of a non-magnetic oxide

**DOI:** 10.1038/ncomms11781

**Published:** 2016-06-10

**Authors:** T. Taniuchi, Y. Motoyui, K. Morozumi, T. C. Rödel, F. Fortuna, A. F. Santander-Syro, S. Shin

**Affiliations:** 1 Institute for Solid State Physics, University of Tokyo, Chiba 277-8581, Japan; 2 CREST, Japan Science and Technology Agency, Tokyo 102-0075, Japan; 3 CSNSM, CNRS/IN2P3 and Université Paris-Sud, Bâtiments 104 et 108, Orsay 91405, France; 4 Synchrotron SOLEIL, L'Orme des Merisiers, Saint-Aubin-BP48, Gif-sur-Yvette 91192, France

## Abstract

Two-dimensional electron gases at oxide surfaces or interfaces show exotic ordered states of matter, like superconductivity, magnetism or spin-polarized states, and are a promising platform for alternative oxide-based electronics. Here we directly image a dense population of randomly distributed ferromagnetic domains of ∼40 nm typical sizes at room temperature at the oxygen-deficient surface of SrTiO_3_, a non-magnetic transparent insulator in the bulk. We use laser-based photoemission electron microscopy, an experimental technique that gives selective spin detection of the surface carriers, even in bulk insulators, with a high spatial resolution of 2.6 nm. We furthermore find that the Curie temperature in this system is as high as 900 K. These findings open perspectives for applications in nano-domain magnetism and spintronics using oxide-based devices, for instance through the nano-engineering of oxygen vacancies at surfaces or interfaces of transition-metal oxides.

## Abstract (web summary)

The surfaces of transition metal oxides exhibit a wide range of functional behaviours, from magnetism to superconductivity. Here, the authors use high-resolution microscopy to image the temperature dependent development of nanoscale ferromagnetic domains on an oxygen-deficient SrTiO_3_ surface.

## Graphical abstract

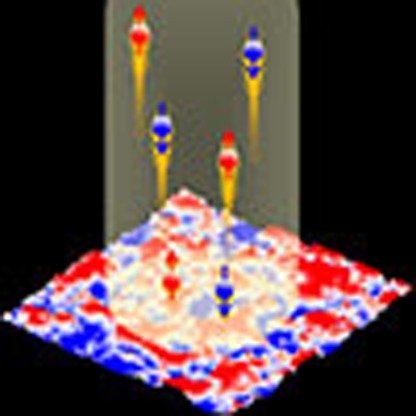

Thanks to their wide range of physical properties in the bulk, and their epitaxial compatibility, transition-metal oxide perovskites provide an ideal arena to explore the competition, interaction and creation of novel ground states at their interfaces^1^,^2^,^3^,^4^,^5^,^6^,^7^,^8^,^9^,^10^,^11^,^12^. The two-dimensional electron gas (2DEG) at the interface between SrTiO_3_ (STO) and LaAlO_3_ (LAO), two wide-gap insulating perovskites in the bulk, is a paradigmatic example of such emerging behaviour. The ground state at the LAO/STO interface can be tuned by external bias gating to show metal-to-insulator transitions^2^, superconductivity below 300 mK (refs 3, 4) or magnetoresistance^5^. Moreover, it was recently demonstrated that the LAO/STO heterostructures also show sub-micrometre patches of ferromagnetism^6^,^7^,^8^, although neither LAO nor STO are magnetic in the bulk. However, it is difficult to verify if such intrinsic magnetism exists only at the interface. Most of the previous studies have used macroscopic probes, such as magnetoresistance, net magnetization or torque magnetometry^5^,^6^,^7^,^8^. In the case of LAO/STO heterostructures, for instance, scanning superconducting quantum interference device microscopy experiments found micrometre-sized ferromagnetic patches in the plane parallel to the interface^7^,^13^,^14^, but the specific depth where the moments reside cannot be determined from this probe. Thus, it is essential to determine the microscopic nature of the observed ferromagnetism: is it intrinsic to the LAO/STO interface? Can it be also found (and under what conditions) at the bare STO surface? Or is it an effect extended over the bulk of STO? More generally, the observation of low-dimensional ferromagnetism in non-magnetic Ti-based oxides would be extremely important both from a fundamental perspective and for its potential in applications in oxide-based electronics^10^,^11^,^12^,^15^,^16^.

Besides the case of the SrTiO_3_-based heterostructures, it was also found that the bare SrTiO_**3**_ surface sustains a 2DEG arising from oxygen vacancies^10^,^11^,^15^, whereas recent experiments indicated the possible existence of magnetism at the surface of SrTiO_3_ (ref. 12). In principle, magnetism in SrTiO_**3**_ could arise from anion defects in the bulk^17^,^18^,^19^,^20^, or could be specific to the surface. Such a surface ferromagnetism would be a leading example of truly emergent phenomena at transition-metal oxide surfaces. In the present work, we investigated the magnetism at the oxygen-deficient surface of SrTiO_**3**_ using laser-based ultrahigh resolution photoemission electron microscopy (laser-PEEM)^21^, a recently developed technique allowing a direct magnetic imaging of a sample surface, even in bulk insulators, with a high spatial resolution of 2.6 nm. Our data show a high concentration of ferromagnetic nano-domains developing below ∼900 K, of sizes around 40 nm at room temperature, at the oxygen-deficient surface of SrTiO_3_. The existence of such nano-domain magnetism up to temperatures well above room temperature is appealing for applications using oxide-based electronics. More generally, given the variety and rich phase diagrams of complex oxides, our results highlight the use of oxide surface states as model systems towards new physics and functional devices.

## Results

### Laser-PEEM magnetic imaging

**Figure 1:**
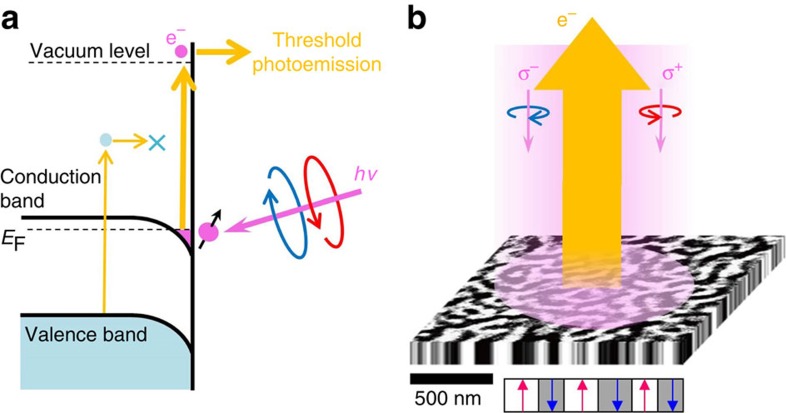
Schematic illustration and demonstration of magnetic imaging. (**a**) Excitation process of threshold photoemission at the surface SrTiO_3_. The photon energy of our laser is 4.66 eV. The band gap of bulk SrTiO_3_ is 3.2 eV. The laser selectively excites photoelectrons derived from the metallic surface. Circularly polarized photons are irradiated to the sample surface, and the spin polarization of the ejected photoelectrons is detected. (**b**) Magnetic imaging technique using ultrahigh resolution laser-PEEM. The magnetic image in the illustration shows a magnetic domain structure in a FePt epitaxial thin film with perpendicular magnetic anisotropy as a typical example. The field of view is 2 μm. The difference between two images taken with left-handed and right-handed circular polarizations (σ^+^ and σ^−^, respectively) shows magnetic contrast. The magnetic contrast originates from magnetization components along the incident direction of photons. The best lateral resolution of our microscope is ∼2.6 nm (ref. 15).

Our magnetic microscopy studies were carried out using magnetic circular dichroism (MCD) imaging of PEEM. Although MCD conventionally requires synchrotron X-rays with circular polarizations^22^, and has been used recently to study the origin of ferromagnetism is LAO/STO heterostructures^23^,^24^, we applied instead the threshold photoemission technique using ultraviolet laser sources^21^. In fact, threshold photoemission laser-PEEM is a powerful tool for observing magnetic domain structures, particularly for metallic magnetic layers on top of bulk insulators. The photoelectron yield in the case of threshold photoemission depends on the work function of the material. For metallic states at the surface of SrTiO_**3**_, the photoemission threshold for the insulating bulk is higher than that for the conductive surface. In our case, the photon energy of our laser source (4.66 eV) is not enough to make photoelectrons from the insulating bulk transcend the vacuum level at the surface, which is of at least 7.4 eV (4.2 eV of work function +3.2 eV of band gap). As a result, these photons excite photoelectrons coming only from metallic surface layers (Fig. 1a). Thus, threshold photoemission is selectively sensitive to the metallic states at the surface. For magnetic imaging, we use right- and left-handed circular polarizations, exploiting the MCD near the Fermi level^25^,^26^. Therefore, PEEM imaging with the threshold photoemission MCD technique is considered to be suitable to investigate ferromagnetism on a thin metallic layer with a small magnetization. Using this technique, we can also determine the direction of magnetization. For instance, by illuminating samples with perpendicular incidence, as indicated in Fig. 1b, the MCD signal exhibits a clear asymmetry, directly related to the magnetization components perpendicular to the surface^21^. The MCD asymmetry is defined here by *A*=(*I*^left^−*I*^right^)/(*I*^left^+*I*^right^), where *I*^left^ and *I*^right^ are the photoelectron intensities at a lateral position in the PEEM images taken with left- and right-circularly polarized lights, respectively.

### Samples

**Figure 2:**
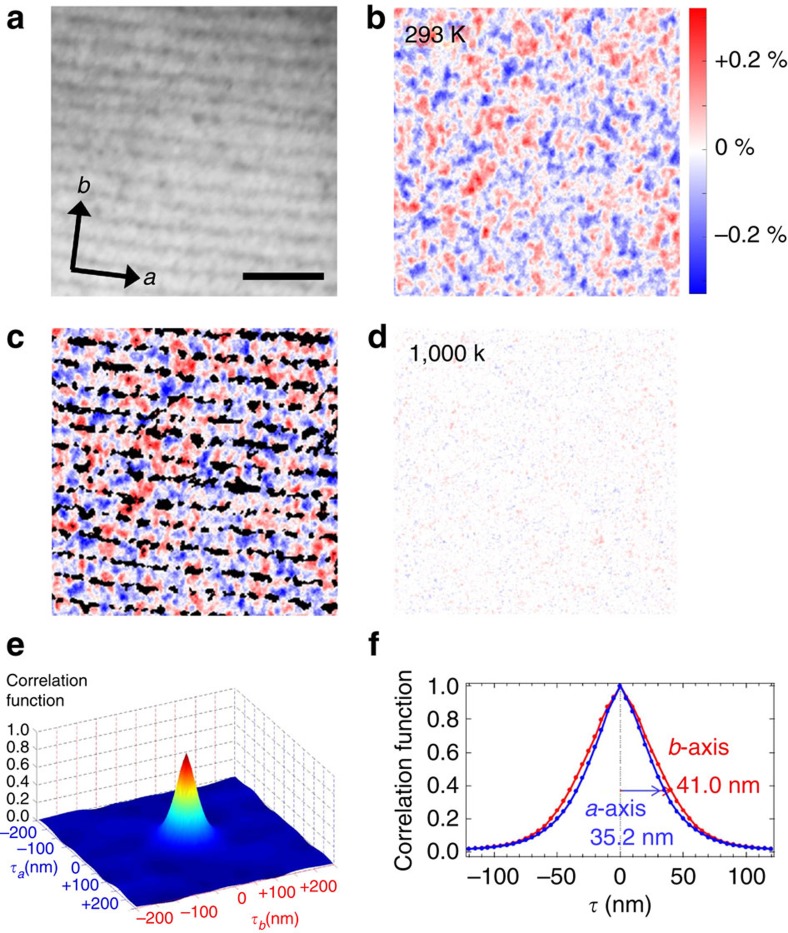
Direct evidence for ferromagnetic nano-domains at the SrTiO_3_ surface. (**a**) Raw laser-PEEM image after annealing at 1,000 K. The black lines in the image correspond to atomic steps. The scale bar at the bottom right of the image corresponds to 500 nm. We confirmed that the terrace width did not change during the annealing by comparison with atomic force microscopy and laser-PEEM images before the annealing, which means that step bunching did not occur. After annealing at 1,000 K, the sample with metallic states at the surface is still transparent after the experiments. (**b**) Magnetic image at room temperature at the same position as the raw image. Red and blue colours indicate positive and negative MCD signals, respectively, which correspond to perpendicular magnetization with spin-up and spin-down. The MCD asymmetry is defined here as *A*=(*I*^left^−*I*^right^)/(*I*^left^+*I*^right^), where *I*^left^ and *I*^right^ are the photoelectron intensities at a lateral position in the PEEM images taken with left- and right-circularly polarized photons, respectively. In these measurements, the light incidence is perpendicular to the sample's surface. With this geometry, the MCD measures the perpendicular components of the magnetization vector. (**c**) Magnetic image overlaid on the step structure from the previous two images. The magnetic domains are partially defined at terraces. (**d**) Magnetic image at 1,000 K. The colour scale of magnetization is the same as in **b**. The magnetic domains have disappeared at 1,000 K. (**e**) Auto-correlation function estimated from the magnetic image of **b**. *τ*_*a*_ and *τ*_*b*_ are the displacement lengths along the *a*- and *b*-axes, as shown in the inset of **a**. (**f**) Auto-correlation function parallel (blue curve) and perpendicular (red curve) to the step edges. Their respective correlation lengths are 35 and 41 nm. The correlation length is estimated as the distance between the origin to the point at 1/*e* height of the correlation function.

For our experiments, we used non-doped TiO_2_-terminated SrTiO_**3**_ (001) single crystals. The crystals were then flash-annealed at 1,000 K for 30–60 s in ultrahigh vacuum (better than 1 × 10^−6^ Pa) to produce oxygen vacancies at the surface^15^,^27^. After annealing, the bulk crystals were still transparent and showed an atomically flat surface with step-and-terrace structure (Fig. 2a). The sample-preparation procedure and characterization are further detailed in the Methods section.

### Laser-PEEM imaging of ferromagnetic nano-domains at the SrTiO_3_ surface

The MCD imaging was performed in the geometry with perpendicular incidence, which detects magnetization components perpendicular to the surface as described above. Figure 2b shows the MCD laser-PEEM image observed at the same position as Fig. 2a. The measurement was performed at room temperature. The image presents clear magnetic-domain structures of opposite MCD contrast, shown by the red and blue coloured areas, indicating perpendicular components of the magnetization with opposite orientations. In fact, the contrast in MCD signal in Fig. 2b should come from the surface, as essentially all photoelectrons come from the metallic surface layer as mentioned above. Moreover, we observe that the magnetic domains are homogeneously distributed over a whole field of view, unlike the previous studies in the LaAlO_**3**_/SrTiO_**3**_ heterostructures using scanning superconducting quantum interference device microscopy^7^, which showed only very localized magnetic signals separated by several micrometres or tens of micrometres. This can also be seen from the magnetic image overlaid on the step pattern extracted from the direct surface image, as shown in Fig. 2c. Note that the magnetic domains are not specifically localized around surface atomic steps, although, as we will see later, the size of the domains is slightly different along and perpendicular to the step edges. All these observations directly demonstrate the existence of intrinsic room-temperature ferromagnetism at the oxygen-deficient surface of SrTiO_3_. As shown in Fig. 2d, the contrast in MCD signal vanishes when measured at higher temperature (∼1,000 K), indicating that it arises from ferromagnetic ordering occurring below 1,000 K. These results were reproduced in samples with different annealing temperatures above 900 K. For samples annealed at lower temperatures, no ferromagnetism was found (see Supplementary Note 1 with Supplementary Fig. 1). One possible reason is that a critical concentration of oxygen vacancies at the surface, obtained only after annealing above 900 K, is necessary to create a magnetic order.

The size of the magnetic domains was estimated using an autocorrelation technique. The autocorrelation function of the magnetic image along a given direction *x*, noted *ϕ*(*x*), was obtained from the cross section of the MCD asymmetry along *x*, *A*(*x*), through the autocorrelation relation:


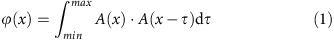


Here *τ* is a displacement length of the magnetic image along *x*, and the integration limits are given by the size of the magnetic image. Figure 2e shows the two-dimensional autocorrelation function calculated from the magnetic image shown in Fig. 2b. *τ*_*a*_ and *τ*_b_ are displacement lengths along the *a*- and *b*-axes defined in Fig. 2a: the directions parallel and perpendicular to the step edges, respectively. Thus, the average domain size is defined as the value of the autocorrelation length *τ* at 1/*e* height of the maximum correlation function, and is estimated from Fig. 2e to be 38 nm. This value is much smaller than the terrace widths of the sample surface (125 nm).

Using this approach, we found that the size of the magnetic domains depends on the atomic step direction at the sample surface. Figure 2f shows the cross-sections of the two-dimensional autocorrelation function along the *a*- and *b*-axes, blue and red lines, respectively. We see that the average correlation lengths are ∼35 nm along the step edges, and 41 nm perpendicular to the step edges. This difference indicates that the observed magnetism originates from the sample surface, as it is correlated to the topography of the topmost atomic layer (see Supplementary Note 1 and Supplementary Fig. 2 for further discussions).

### Temperature evolution of the ferromagnetic nano-domains

**Figure 3:**
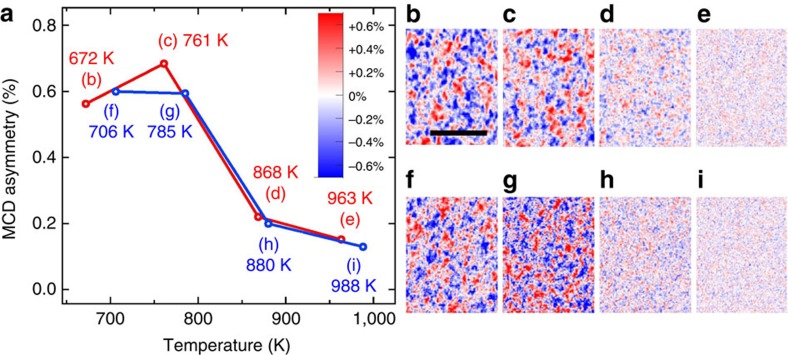
Temperature dependence of the magnetic contrast. (**a**) The asymmetry in the out-of-plane MCD signal, extracted from the acquired MCD images, is plotted as a function of measurement temperature. The samples were first annealed at 1,000 K. (**b**–**e**) and (**f**–**i**) Perpendicular magnetic domain structure images for the indicated temperatures in **a**, which were measured by the Laser-PEEM with threshold photoemission MCD. The scale bars correspond to 1,000 nm. From the evolution of the MCD contrast, the Curie temperature is estimated to be around 900 K. The red and blue lines in panel **a**, and their associated MCD images in panels **b**–**e** and **f**–**i**, respectively, correspond to two independent sets of measurements.

To determine the Curie temperature of this system, we performed the magnetic imaging at different temperatures. As mentioned previously, the samples were annealed at 1,000 K and subsequently measured at different temperatures with perpendicular light incidence. The results are shown in Fig. 3. The asymmetry in the perpendicular MCD signal, extracted from the MCD laser-PEEM images, is plotted as a function of temperature in Fig. 3a. Figures 3b–i are MCD laser-PEEM images for the indicated temperatures. The red and blue contrasts show the magnetization components perpendicular to the surface. Based on these results, the estimated Curie temperature for ferromagnetic ordering is around 900 K. These measurements were reproducible over different samples.

### Response to an external perpendicular magnetic field

**Figure 4:**
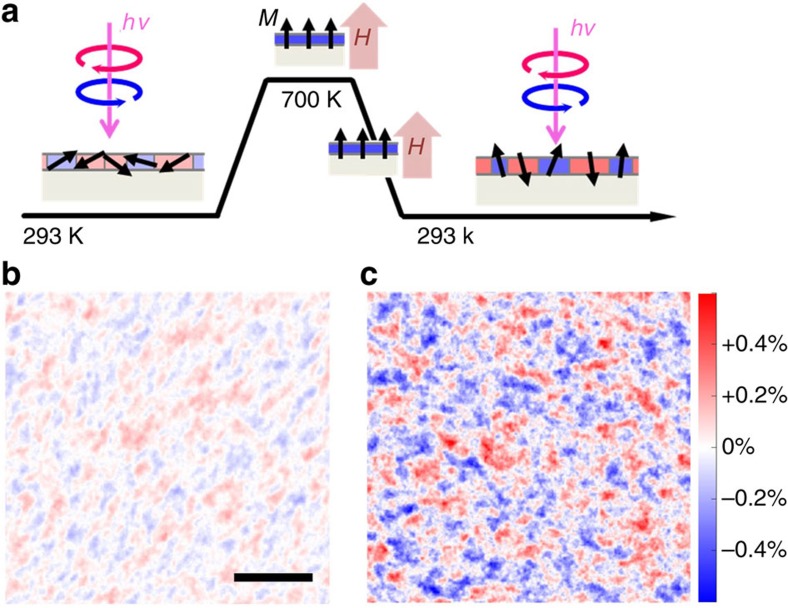
Magnetic domain formation before and after application of an external magnetic field. (**a**) Procedure of measurements. The geometry of the magnetic imaging is sensitive to the perpendicular component of the magnetization. The sample was first flash annealed at 1,000 K, and then measured at room temperature. Afterwards, the sample was heated up to 700 K to lower its coercivity. A magnetic field of about 1,000 Oe perpendicular to the sample surface was applied at 700 K, and maintained during cooling down to room temperature. The sample was finally measured in zero field at room temperature. (**b**) Magnetic image of an as-annealed sample before application of the external magnetic field. The scale bar corresponds to 1,000 nm. (**c**) Magnetic image of the same sample after the magnetic field was applied. The magnetic images are shown using the same colour scale. The magnetic contrast has increased by applying the magnetic field as the magnetization vectors of the different magnetic domains are tilted towards the sample normal.

To confirm the magnetic origin of the MCD domains, we measured the change of the MCD contrast after application of an external magnetic field. The experimental procedure is shown in Fig. 4a: we performed the MCD imaging in an as-annealed sample (Fig. 4b). Then the sample was heated up to 700 K in ultrahigh vacuum while applying a perpendicular magnetic field of ∼1,000 Oe from a commercial samarium-cobalt magnet held in front of the sample. After cooling to room temperature in magnetic field, we carried out again an MCD imaging at room temperature and zero field, as shown in Fig. 4c. The increase of the MCD contrast after the magnetic field was applied is evident by comparing Fig. 4b,c. Such a response to the magnetic field clearly shows that the image contrast between different domains comes from their magnetism. More precisely, as illustrated in Fig. 4a, our results show that before applying the external magnetic field, the magnetization vectors are not saturated along the sample normal but tilted (see discussion in the Supplementary Note 2 and Supplementary Fig. 3). After applying the external field, the perpendicular component increases because of the large perpendicular magnetic anisotropy. In Fig. 4c, we did not observe a preferential alignment of the magnetic moments (anti-)parallel to the field, as evidenced by the similar areas covered by the red and blue domains. Applying the field in the opposite direction (−1,000 Oe, not shown) yields identical results.

To explain these observations, we first discuss the initial state of our system, Fig. 4b. Owing to the competition between the perpendicular magnetic anisotropy and the in-plane shape anisotropy, which tends to reduce the demagnetization energy derived from the film-like shape of the magnetic layer, the initial state shows various magnetization distributions: some are in-plane, other out-of-plane or tilted. The application of a perpendicular magnetic field above the Curie temperature results in a single domain with magnetization perpendicular to the surface under the field. After switching off the field at room temperature, the magnetostatic energy of the total system is reduced by flipping some of the moments and thereby, breaks the single domain state. Such a behaviour often happens, especially in very thin films^28^.

## Discussion

Our results indicate that ferromagnetism in SrTiO_3_ is closely related to oxygen vacancies. In this material, oxygen vacancy formation results in the creation of itinerant electrons, as well as localized electrons and magnetic moments^29^,^30^. At interfaces or surfaces, the interaction between itinerant and localized electrons determines the magnetic state (refs 31, 32, 33). The interplay between these two types of electrons can lead to rich magnetic phase diagrams not only in SrTiO_3_-based 2DEGs^34^,^35^, but also in diluted magnetic semiconductors^36^,^37^. Using the MCD laser-PEEM technique, neither the concentration of localized nor the one of itinerant electrons can be directly accessed. Hence, the microscopic mechanism for the creation of the magnetic moments cannot be deduced from our data. However, the measured high Curie temperature (*T*=900 K) can exclude RKKY interaction as the mechanism responsible for the onset of magnetism in the 2DEG at the surface of SrTiO_3_, because the indirect exchange coupling between impurity moments in semiconductors via the RKKY interaction is very weak and forms ferromagnetic ordering at only very low temperatures (*T*<20 K)^37^.

Our finding of ferromagnetic nano-domains at the surface of a non-magnetic oxide presents a playground for the engineering of innovative nano-devices with properties suitable for applications in oxide electronics and spintronics: high ferromagnetic transition temperature, conductive surface, highly insulating bulk matrix entailing optical transparency, negligible leakage currents, higher breakdown voltages, lower electronic noise and high-temperature and high-power operation.

## Methods

### Sample preparation and characterization

We used non-doped SrTiO_**3**_ (001) substrates that were TiO_2_ terminated by a treatment in a buffered HF solution and subsequent annealing^15^,^16^. The purity of the crystals was 99.9866 %. The main components of impurities were Ba (129 p.p.m.) and Ca (5.4 p.p.m.). Components of transition metal elements were under detection limit: for example, Cr (<2 p.p.m.), Cu (<10 p.p.m.), Fe (<1 p.p.m.), Mn (<0.2 p.p.m.) and Ni (<5 p.p.m.). An atomically smooth surface with clear unit-cell-height steps was confirmed by atomic force microscopy. The SrTiO_**3**_ crystals were then flash-annealed by rising their temperature up to 1,000 K during 10 min, and then keeping them at that temperature in ultra-high vacuum for 30–60 s to produce a metallic state with oxygen vacancies at the surface. The samples were heated from the backside using electron bombardment to avoid any adsorption of contaminants on the surfaces. The side to be measured was isolated from the backside to be heated by a mask made of non-magnetic molybdenum. After annealing, the bulk crystals were still transparent and showed an atomically flat surface with step-and-terrace structure (Fig. 2a).

### Laser-PEEM MCD imaging

Our PEEM instrument has electron mirror optics and an energy analyser to reduce spherical and chromatic aberrations in the electron optics^21^. The best lateral resolution of the microscope is 2.6 nm. Threshold photoemission MCD, which we applied in this study, usually detects spin polarizations near the Fermi level around the Γ point of the sample surface. Therefore, this technique is especially suitable for samples with single crystalline surfaces, like SrTiO_**3**_. The wavelength of the laser we used is 266 nm, which corresponds to a photon energy of 4.66 eV. A photo-elastic modulator was used to switch between left- and right-handed circular polarizations. If we use intense pulsed light sources, which are conventionally employed for magnetic imaging, the lateral resolution of PEEM significantly degrades due to the Coulomb repulsion between the ejected photoelectrons. To avoid such a space charge effect, we used a continuous wave laser as an excitation source. The intensity of the laser is 2 × 10^18^ photons per second. In spite of using such an intense laser, we did not see any deterioration of the resolution. The combination of very intense continuous wave laser and polarization-switching method enables us to precisely measure even a small MCD asymmetry with high lateral resolution. The accuracy of the MCD measurement is ∼0.1 %, which was estimated from the noise level of Fig. 2d showing no MCD signal above the Curie temperature. MCD signals correspond to the magnetization direction projected to the incident direction of the light source. Therefore, if the illumination angle is perpendicular to the sample surface, this geometry shows magnetic components perpendicular to the surface. As the path of photoelectrons in our microscope is bent by a magnetic field after they pass through a hole of objective lens in front of the sample surface, we are able to perform the perpendicular incidence measurements without causing interference with components of the microscope. For the details of experimental geometry, see the Suuplementary Note 3 with Supplementary Fig. 4 and (ref. 21).

We did not observe any effect of the laser irradiation on the sample surfaces. If the number of oxygen vacancies increased with the laser irradiation, we would be able to observe it as an increase in photoelectron intensity because of the increase in the number of Ti 3*d* electrons (carriers) in the conduction band, as shown in Fig. 1a. However, we never observed any change in photoelectron intensity induced by laser irradiation. Note, furthermore, that the energy of our laser is very small compared with the threshold energy (∼38 eV) necessary to efficiently create oxygen vacancies upon irradiation^38^, whereas its photon flux is much smaller than the one of pulsed lasers.

## Additional information

**How to cite this article:** Taniuchi, T. *et al*. Imaging of room-temperature ferromagnetic nano-domains at the surface of a non-magnetic oxide. *Nat. Commun.* 7:11781 doi: 10.1038/ncomms11781 (2016).

## Supplementary Material

Supplementary Information Supplementary Figures 1-4 and Supplementary Notes 1-3
