# Supplementary material for: Imaging of room-temperature ferromagnetic nano-domains at the surface of a non-magnetic oxide
Source: Nat Commun. 2016 Jun 10;7:11781. doi: 10.1038/ncomms11781 (PMC4906390; doi:10.1038/ncomms11781)
Supplement: Supplementary Information — Supplementary Figures 1-4 and Supplementary Notes 1-3 [file ncomms11781-s1.pdf]

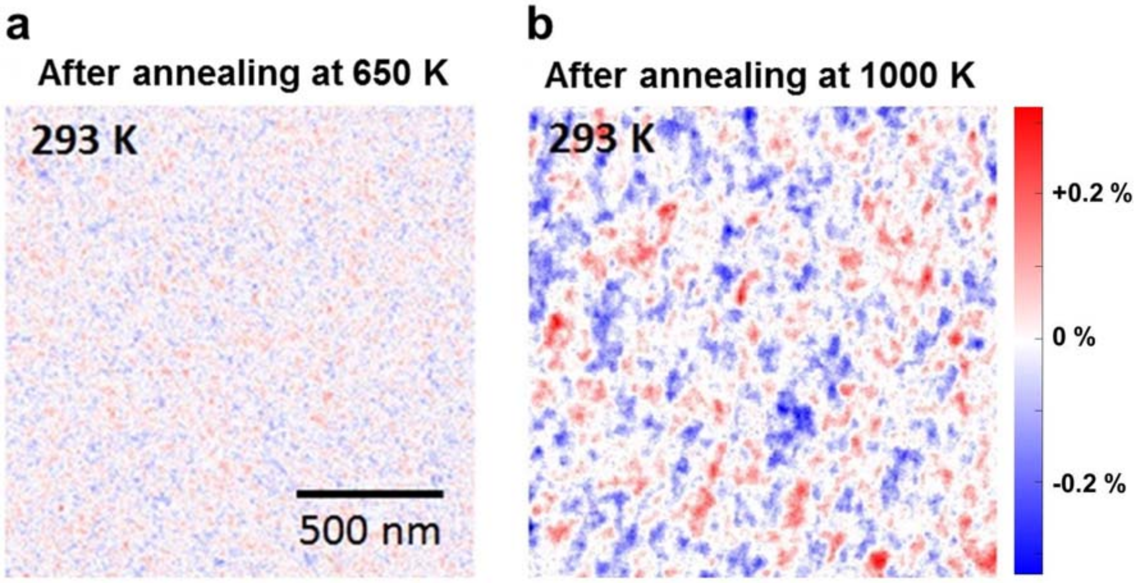

Supplementary Figure 1: Annealing temperature dependence of the magnetic domain formation.

The sample was heated for 1 min in ultrahigh vacuum at (a) 650 K and (b) 1000 K. The magnetic images were measured at room temperature. The incident angle of the laser was perpendicular to the sample surface, which is sensitive to the perpendicular components of the magnetization.

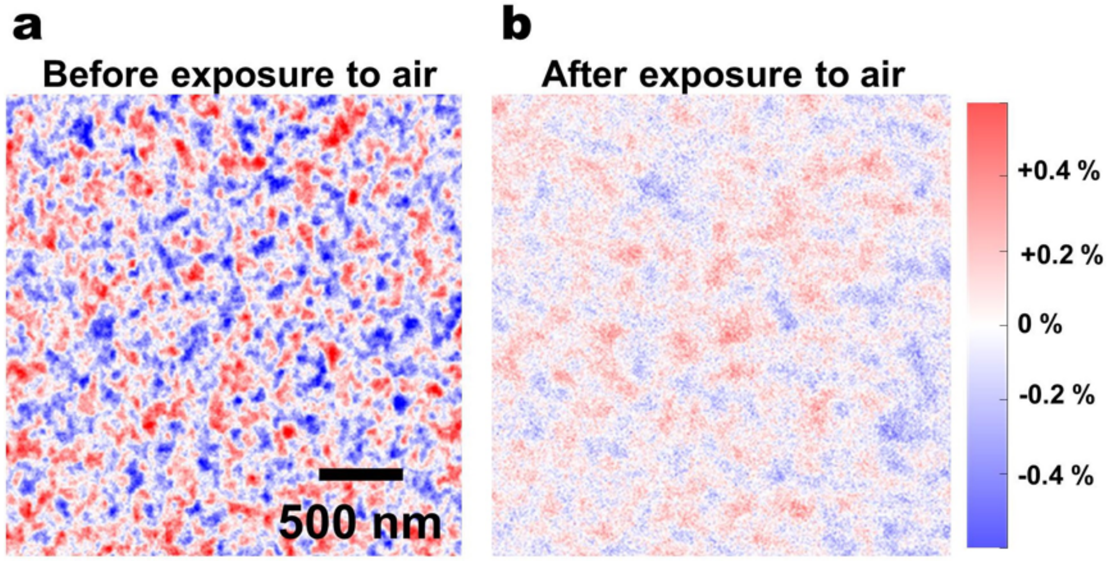

Supplementary Figure 2: Effect of exposure to air on the magnetic domain

formation.

The sample was heated for 1 min in ultrahigh vacuum at 1000 K. (a) Magnetic image taken just after heating, then the sample was exposed to air for 30 seconds. (b) Magnetic image after exposure to air. The measurements were done at room temperature. The MCD asymmetry of the images is 0.55% and 0.25%, respectively.

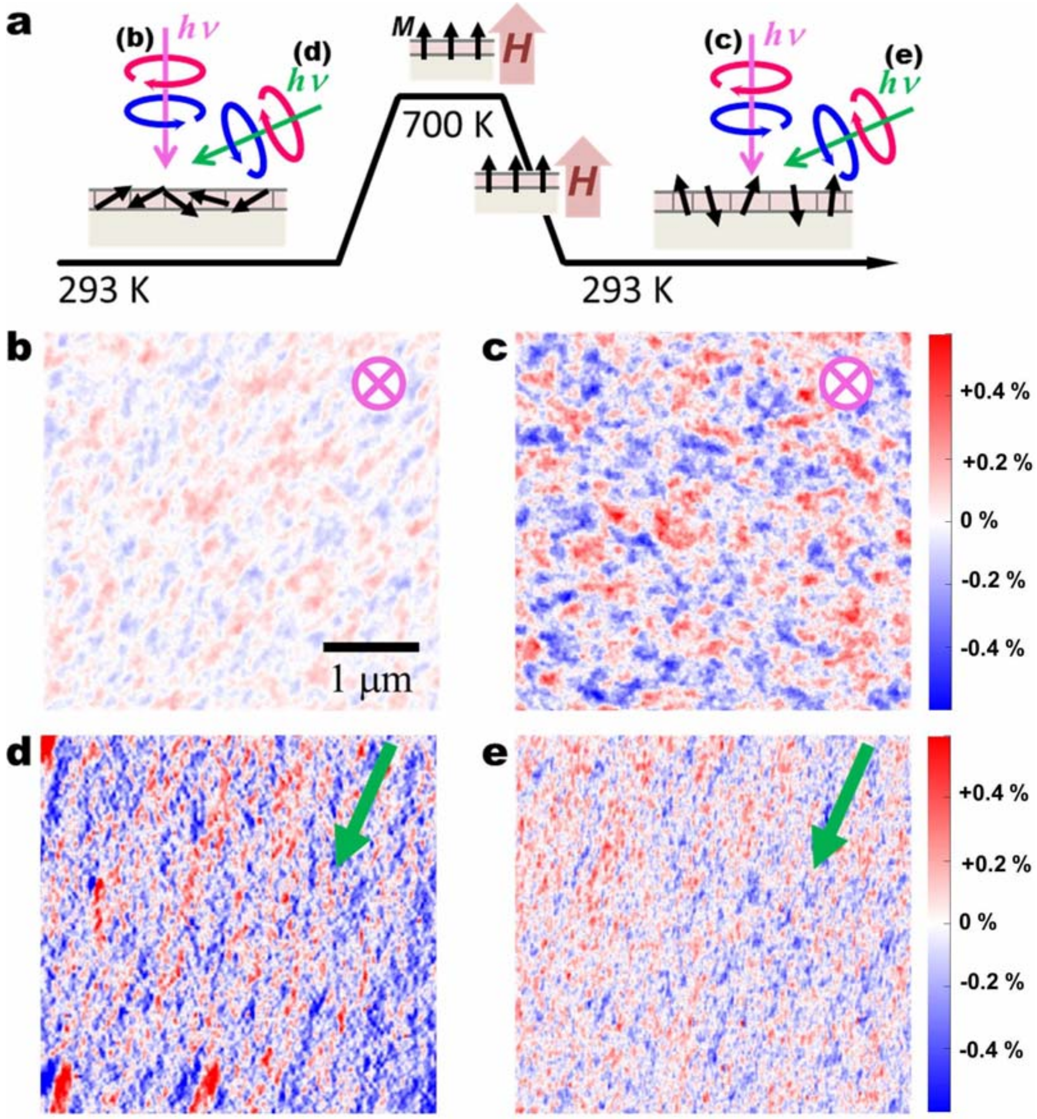

Supplementary Figure 3: In-plane and perpendicular magnetic domain formation before and after application of an external magnetic field. (a) Procedure of measurements and application of magnetic field. The magnetic imaging was performed in geometries sensitive to both in-plane and perpendicular magnetizations. The sample was heated up to 700K to lower its coercivity. A magnetic field, perpendicular to the

sample surface, was then applied and maintained while cooling down to room temperature. All measurements were done under zero field at room temperature. (b, d) Dependence of the magnetic contrast on the angle of incidence of light before the magnetic field was applied. The angles of incidence, marked by arrows, are perpendicular and grazing, respectively. (c, e) Dependence of the magnetic contrast on the angle of incidence of light after application of an external the magnetic field. All the magnetic images are shown using the same color scale.

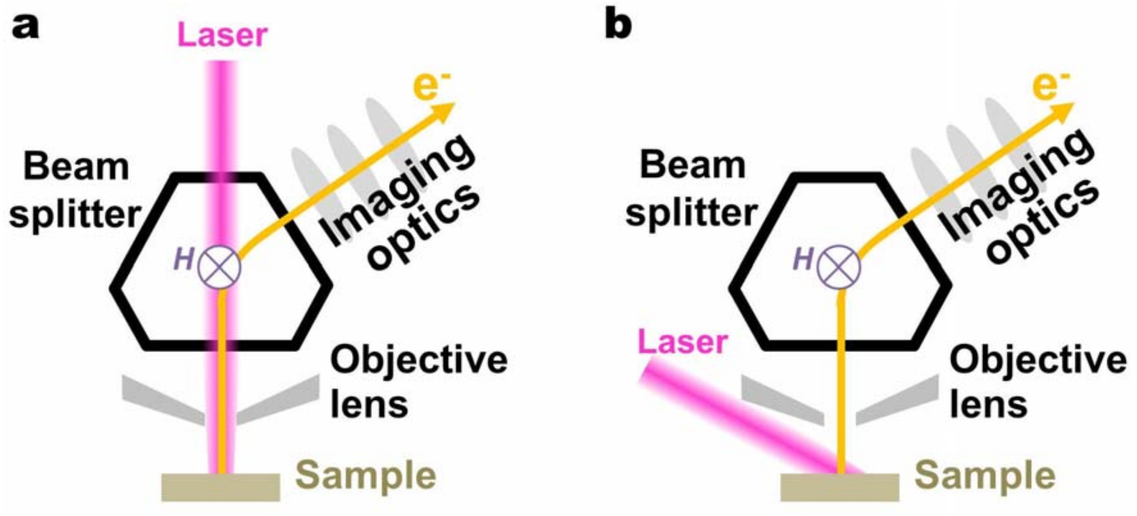

Supplementary Figure 4: Schematic view of the measurement geometry of the microscope. The samples are illuminated with a laser beam with (a) perpendicular incidence and (b) oblique incidence. Photoelectrons are captured by the objective lens. For the perpendicular incidence, the ultraviolet laser, introduced into the PEEM chamber, passes through a beam splitter and the transmission hole in the objective lens. In order to separate the electron beam from the laser beam, the path of the photoelectrons is bent by a magnetic field in the beam-splitter. For oblique incidence, the incident angle of the laser is 16° from the sample surface, and does not need to be separated from the beam of photo-emitted electrons.

Supplementary Note 1: Ferromagnetism

Supplementary Figure 1 shows the magnetic images of the $SrTiO_3$ sample annealed in ultrahigh vacuum at two different temperatures: 650 K and 1000 K. In both cases, the annealing time was 1 min, and the laser-PEEM measurements were done at room temperature (293 K). As shown in Supplementary Figure 1(a), the sample that was annealed at 650 K did not show clear ferromagnetic signals at room temperature. On the other hand, we found magnetic domain formation in the sample annealed at 1000 K. Note that if there were magnetic particles or impurities on the sample's surface, the magnetic contrast would decrease, or at least would not show any changes, by the increase of annealing temperature, simply because of the diffusion of magnetic elements from surface to bulk. Therefore, such a clear difference in magnetic signals can be considered to arise by the increase of oxygen vacancies near the surface. Besides, as shown in Supplementary Figure 2, we also found that the magnetic structures and the magnetic contrast immediately change after the surface is exposed to air, thus providing additional evidence that the magnetism originates from oxygen vacancies near the surface.

Supplementary Note 2: Three-dimensional magnetic anisotropy at the $SrTiO_3$ surface

In the main text, we evoked the perpendicular magnetic anisotropy on the $SrTiO_3$ surfaces. In particular, in Fig. 4 of the main article, we examined the response of the sample to the application of an external magnetic field. Here we discuss in more detail the 3D magnetic anisotropy of the oxygen-deficient $SrTiO_3$ surface.

In laser-PEEM, the MCD contrast reflects the components of the magnetization vector projected along the incident direction of the laser: perpendicular incidence reveals the magnetization components perpendicular to the surface, while grazing incidence is sensitive to in-plane components. Thus, we investigated the in-plane magnetization components at the surface of $SrTiO_3$ by switching the incidence angle of the laser beam. In our setup, the angle in the grazing incidence is 16° to the sample surface. Therefore, the MCD signal in grazing incidence includes also perpendicular magnetic components.

Supplementary Figure 3(a), similar to Fig. 4a of the main text, shows the experimental procedure: after a first set of MCD measurements at room temperature, we heated the sample up to 700 K in ultrahigh vacuum, then applied a magnetic field perpendicular to the surface. After cooling to room temperature with the field, we carried out again imaging under zero field conditions. Supplementary Figures 3(b) and 3(d) are magnetic images of the sample as annealed, which were measured with perpendicular and grazing incidences, respectively. Supplementary Figures 3(c) and 3(e) show magnetic images after applying the field with perpendicular and grazing incidences. The red-white-blue colors in all the MCD images are normalized to the same scale. As shown by the images, magnetic contrast is observed for both directions of incidence. This implies that the magnetization orientation is tilted away from the surface normal. Note that the existence of magnetization components perpendicular to the surface strongly indicate a large magnetic anisotropy, as the very thin magnetic metallic layer at the surface of $SrTiO_3$ needs to overcome the very large shape anisotropy, where magnetizations tend to align in-plane.

In threshold photoemission MCD measurements, it is difficult to compare quantitatively the perpendicular and in-plane magnetization components, and thus determine the degree of magnetization tilting. This would require a precise knowledge of the contribution to the MCD signals of both the incident and reflected light at the sample surface. But on grazing-incidence geometry, the MCD signal of the reflected light counters the MCD signal due to the incident light (see Ref. [21] of the main text). Nevertheless, we can discuss the existence of perpendicular magnetic anisotropy by comparing the changes in MCD contrast before and after applying a magnetic field. Supplementary Figures 3(c) and 3(e) show magnetic images with perpendicular and grazing incidences after the external magnetic field was applied. Comparing the color scale of perpendicular and grazing MCD contrasts between Supplementary Figures 3(b) and 3(c), and Supplementary Figures 3(d) and 3(e) respectively, we find an increase of perpendicular components and a concomitant decrease of in-plane components after the field was applied. Such changes provide further evidence of the large perpendicular magnetic anisotropy at the oxygen-deficient surface of $SrTiO_3$. Note that when the magnetic field was applied to the sample, the sample was instantly heated up to 700 K to assist the magnetization flip. During this procedure, more oxygen vacancies may have been generated on the sample surface, resulting in an increased MCD signal. However, as seen in Supplementary Figures 3(d) and 3(e), we found a significant decrease in the in-plane MCD signal after the procedure of heating + applying a

magnetic field perpendicular to the surface + cooling under field. This indicates that the heating to 700 K has actually no significant effect on the creation of oxygen vacancies.

Supplementary Note 3: Microscope

A schematic illustration of the measurement geometry for our laser-PEEM system is shown in the Supplementary Figure 4. The light is focused into a beam of 40 μm diameter. Samples are floated up to –20 kV to accelerate the photoelectrons between the sample surface and the objective lens. The beam splitter uses a magnetic field to separate the path of the photoelectrons from the laser path. For oblique incidence, the incident angle of the laser is 16˚ from the sample surface, so that MCD signals for oblique angle also include perpendicular components of the magnetization vector. A thorough description of the laser-PEEM setup can be found in Ref. [21] of the main text.
